# A century of *Azospirillum*: plant growth promotion and agricultural promise

**DOI:** 10.1080/15592324.2025.2551609

**Published:** 2025-08-27

**Authors:** Ramón Pelagio-Flores, Gustavo Ravelo-Ortega, Ernesto García-Pineda, José López-Bucio

**Affiliations:** a Facultad de Químico Farmacobiología, Universidad Michoacana de San Nicolás de Hidalgo, Morelia, Mexico; b Secretaría de Ciencias, Humanidades, Tecnología e Innovació, Morelia, Mexico; c Instituto de Investigaciones Químico-Biológicas, Universidad Michoacana de San Nicolás de Hidalgo, Morelia, Mexico

**Keywords:** *Azospirillum*, growth promotion, nutrient acquisition, agriculture, auxin

## Abstract

The genus *Azospirillum* celebrates 100 y since its discovery in 1925 by Martinus Willem Beijerinck, who worked with *Spirillum lipoferum* as a starting species. Decades of work involving laboratory and field research endorse their various beneficial properties, such as plant rooting, mineral nutrition, hormonal strengthening, and the activation of cellular and molecular responses, which lead to better growth, development, and productivity. Some hormones, such as auxins and cytokinins, potentiate root branching through their effects on mitosis, and via signal transduction mediated by the Target Of Rapamycin (TOR) kinase. Although initial efforts were aimed at clarifying the importance of biological nitrogen fixation in plant growth in the face of root colonization with *Azospirillum*, recent advances show that these bacteria also activate the mechanisms of acquisition of phosphorus and iron, two essential nutrients for fulfilling the plant's life cycle. In recent years, *Azospirillum* structural elements such as flagellin and lipopolysaccharides emerged as elicitors, influencing the development and defense of the host. Goals have also been achieved in formulating biotechnological products, whose application has increased in countries such as Argentina and Brazil, showing relevant and promising results toward saving fertilizer, optimizing management, and ultimately, making agriculture more sustainable.

## Introduction

Over the years, plant growth-promoting bacteria of the genus *Azospirillum* have been consolidated as an important alternative for agriculture. An initial survey in African countries, Brazil, and the United States notoriously found the bacteria were more associated with the roots of cereal and forage grasses, which led to the establishment of the *Azospirillum* genus with two former species, *A. lipoferum* and *A. brasilense.*^1^^,^^2^ Current advances have demonstrated the cosmopolite distribution of *Azospirillum,* with many new species being discovered every year.^3^

**Figure 1.**
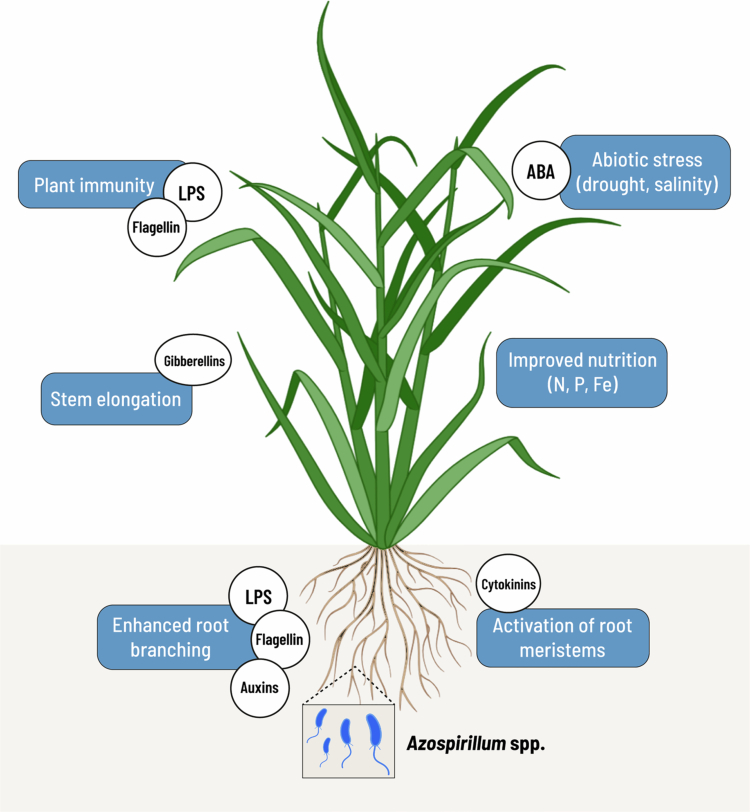
Mechanisms of plant growth promotion by *Azospirillum* spp. Bacterial crop inoculation increases tolerance to abiotic stress through abscisic acid-mediated osmotic adjustment, and enhanced root growth and branching involve the secretion of auxin and cytokinin, as well as structural cell components such as flagellin and lipopolysaccharides (LPS) that act as elicitors also influence host immunity. Stem elongation may involve bacterial gibberellin detection. Overall biomass enhancement occurs through biological nitrogen fixation as well as mechanisms for improved phosphorus and iron acquisition.

*Azospirillum* spp. are gram-negative bacteria that move through single or multiple flagella. Flagellin is the main protein configuring the flagellum and is a major elicitor for plant immunity.^4^ Genome analysis led to the reclassification of *A. brasilense* Sp245 as the type strain of *Azospirillum baldaniorum* sp. nov.^5^ and *A. brasilense* Az39 as the type strain of *Azospirillum argentinense* sp. nov.^6^ The agronomical relevance of *Azospirillum* is increasing, as it is extensively applied for plant growth promotion given the lack of deleterious effects and owing to its many beneficial traits.^7^^,^^8^ This is due to the colonization of a large number of plant species, structural cellular components such as flagellin and lipopolysaccharides (LPS) that act as plant elicitors, the production of hormones that modify the physiology of plants, their capacity to fix nitrogen and configure root architecture, which enables more efficient acquisition of micro and macronutrients, and the possibility of being applied to the root or the foliage with consistent results (Figure 1).

Excellent reviews have been written by leading experts in the field describing major advances over the years whose impact has been undeniable in recognition of *Azospirillum* spp. as beneficial plant bacteria.^7,9–13^ The aim of this study is not to revisit those classic works but instead pose the challenge of integrating agronomical and experimental experiences and present a synthesis into the current knowledge about the mechanisms of plant growth promotion by *Azospirillum* spp.

## Development of *Azospirillum* as a promising plant probiotic

The application of *Azospirillum* spp. represents an exceptional case of translational biology, in which the use of a microorganism, in this case, a bacterium, first showed promising potential in the field to boost crop growth and productivity, and over time, the different facets of this beneficial behavior were scrutinized. Bashan and de-Bashan,^14^ argued that there is no single mechanism to explain all the potential that a single bacterial isolate has to enhance the growth of its hosts, but several mechanisms that in combination, could exert a synergistic or additive effect, which could be manifested depending on the *Azospirillum* species, their plant host, growth conditions, variations in the environment, as well as the ecological context of the interaction.

The focus on *Azospirillum* was initially microbiological regarding its potential to fix nitrogen, and later on, the possibility of formulating plant biostimulants became a reality. Brazil and Argentina have been leading countries not only in research involving *Azospirillum* but also in its biotechnological application as crop inoculants. In 2009, two products based on *A. brasilense* Ab-V5 and Ab-V6 strains were registered in Brazil with an application of around 10.5 million doses for grasses, including corn, wheat, rice, and *Brachiaria* pastures, and coinoculation of legumes, such as soybean and common bean, which yielded positive effects on root growth, biomass production, grain yield, uptake of nutrients and water, and increased tolerance to abiotic stress.^15^

*Azospirillum brasilense* Az39, recently classified as *A. argentinense* was isolated in the 1980s in the province of Córdoba, Argentina, and used by the bioinoculant industry, mainly for corn, wheat, soybeans, and intensive crop species. Advances have been made to understand how this bacterium is genetically constituted, having numerous mechanisms of interaction with plants, with a strong impact on the development and functionality of the root, making the use of resources by the plant more efficient, but also through nitrogen fixation and hormone production, both of which have very important connotations over crop performance.^16^

Farmer experience using *Azospirillum* spp. indicated that the average yield ranges from 5% to 15% with a success rate of around 80%, and in recent years, both fertilization and inoculation together appears to be a strategy to boost productivity with very promising estimations of saving up to 25% of *N* fertilizer, optimizing investment of up to $15 per Ha, decreasing the emission of *N*-related warming gases, and increasing carbon assimilation and retention.^7^

## Inoculation of *Azospirillum* in the model plant *Arabidopsis thaliana*

Despite being *Azospirillum* spp. ideal partners for crops, the advent of *Arabidopsis*, a small weed for laboratory studies, has enabled a deep understanding of the molecular mechanisms by which plants react to the bacterium, basically due to the massive sequencing techniques developed at the end of the past century, which made possible to know its nearly 25,000 genes.​​​​​​^17^ Meanwhile, transformation techniques to overexpress selected genes and to study their regulation through the fusion of promoters to reporter genes were applied, such as those encoding *β*-glucuronidase and the green fluorescent protein (GFP) from the jellyfish *Aequorea victoria.*^18^ These advances allowed a deep understanding of the *Arabidopsis*–*Azospirillum* interaction, which has opened a new era in the knowledge of the mechanisms for mutual plant–microbe recognition.^19–22^

The development of an *in vitro* system to study the *Arabidopsis*–*Azospirillum* interaction was possible thanks to the use of Petri plates containing transparent agar-solidified media in which a carbon source, usually sucrose, and the nutrients required for both plant and bacterial growth are supplemented.^23^ The Murashige and Skoog formulation was adopted for the growth of *Arabidopsis* under axenic conditions, which also enabled the formation of *Azospirillum* microcolonies since organic acids exuded by roots probably enriched the medium or simply because *Azospirillum* can use sucrose as a carbon source.^20^^,^^21^

The small size of *Arabidopsis* allows growth in inclined Petri dishes for periods of up to 15 d, and the transparent medium facilitates detailed observation of the root system, including the primary root, lateral roots, and root hairs with the help of a stereoscopic microscope.^24^ Three interaction methods have been designed. First, divided Petri dishes are used, with a plastic separation at the center that prevents the diffusion of molecules through the medium. The plant and the bacteria are then placed on opposite sides of the Petri plate, and communication is allowed through the emission of volatile organic compounds that spread in the sealed atmosphere.^25^

The second method of interaction involves the germination of *Arabidopsis* seeds forming a row at one end of the Petri dish. Once the seeds germinate, the plates are tilted vertically to allow the growth of the plant on the surface of the nutrient medium, and a streak is made with the bacterial inoculum at a defined distance (i.e., 3 or 5 cm) from the plant growth site. This system allows the analysis of root growth in response to the diffusible compounds emitted by bacteria, such as hormones and quorum-sensing autoinducers.^26^

In the third method, a streak of the bacterium is sown for 2 or 3 d on the surface of the medium, and then plants are transferred about 4 d after germination, placing the root directly over the streaked inoculum, and both the growth of the bacteria and the plants in interaction can be monitored for several days.^27^ This last system enables direct contact and spread of bacteria throughout the root during its growth, helping in the colonization of the meristems, lateral roots, and root hairs; thus, the plant can recognize structural elements of the bacterium, such as the flagellum and cell wall lipopolysaccharides that act as elicitors of development and defense. In recent years, the Petri plate assay has been highly valuable for assessing the response of plants not only to *Azospirillum* but also to compare their mechanisms of action to other plant growth-promoting rhizobacteria.^28–32^

The *in vitro* system demonstrated its value in the characterization of a large number of bacterial isolates distinguishing plant growth-promoting bacteria from potential pathogens such as *Pseudomonas aeruginosa* PAO1, *Enterobacter* sp., and *Pseudomonas lurida* that halt plant growth just a few hours after inoculation.^31^^,^^33^^,^^34^

Beneficial bacteria notoriously promote the formation of foliage and lateral roots, without compromising primary root growth, such as *Bacillus* sp.^29^ and *Burkholderia phytofirmans* PsJN.^35^ However, a fairly defined group breaks the apical dominance of the taproot, which enhances the development of lateral roots by activating their meristems, such as *Micrococcus luteus,*^36^ *Pseudomonas brassicae,*^31^ *P. putida* and *P. fluorescens,*^26^ and *Azospirillum* spp.^20–22^ The formation of a more dense and shallow root system is believed to facilitate nutrient acquisition, mainly phosphorus and micronutrients, which easily precipitate out of the soil solution and tend to accumulate in the upper soil layers. Therefore, no signs of deleterious plant effects can be appreciated upon root colonization of *Azospirillum* for various days.

## Mechanisms of plant growth promotion by *Azospirillum*

*AzospirilIum* spp. colonize and promote the growth of plants from the Poaceae (Gramineae), Solanaceae, Fabaceae, Cucurbitaceae, Cactaceae, Chenopodiaceae, Convolvulaceae, Fagaceae, and Brassicaceae, indicating that their beneficial effects are not plant specific.^9^^,^^37^

Plant growth promotion induced by *Azospirillum* is multifactorial, and several mechanisms act concertedly to boost biomass production, including the promotion of root branching, nitrogen fixation, mineral nutrient acquisition, phytohormone production, and elicitors such as lipopolysaccharides and flagellin. These strategies help plants not only to take advantage of available resources, such as the momentary availability of nutrients or the response to chemical fertilizers or organic amendments in the case of agricultural ecosystems, but also allow them to adapt to adverse environmental situations. Next, we describe how plants react to the presence of *Azospirillum*.

### Configuration of root architecture

**Figure 2.**
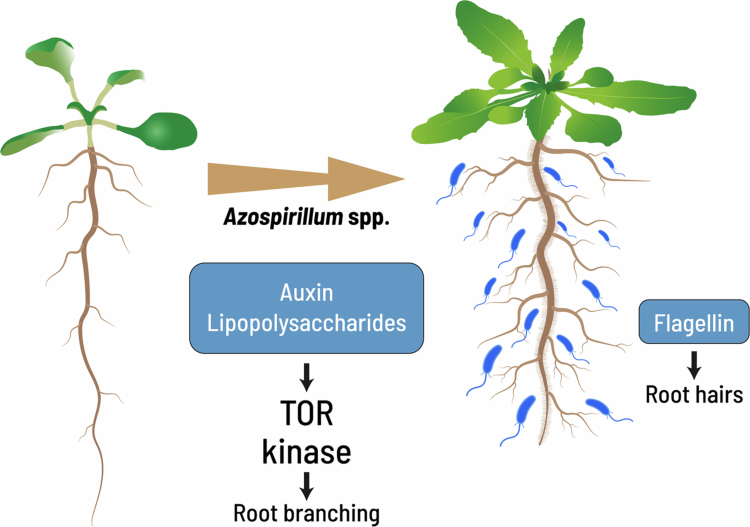
* Azospirillum* auxin and elicitors configure root architecture. Bacterial auxin secretion and lipopolysaccharides inhibit primary root growth while promoting the formation of lateral roots and root hairs through a pathway involving the Target Of Rapamycin (TOR) signaling. On the other hand, flagellin, the major protein from the flagellum, may act to promote root hairs through an as yet unknown mechanism.

Major root architectural changes induced by *Azospirillum* spp. include the inhibition of root growth and the promotion of root branching and root hair development (Figure 2).^20–22,38^

The first work focusing on root developmental responses to *A. brasilense* Sp-5 was carried out by Dubrovsky et al.^19^ The authors examined the growth of root hairs, epidermal cells specialized in water and nutrient uptake in axenic and inoculated plants. A two-fold increase in the total length of root hairs was found in plants inoculated with the bacterium, which was independent of nitrate and carbon application, which led the authors to propose that a hormonal mechanism rather than a nutritional response could be responsible for the enlargement of the root hairs.

Spaepen et al.^22^ compared the root architectural changes in *Arabidopsis* seedlings upon inoculation with *A. brasilense* Sp245 and the FAJ0009 mutant, which lacks indole pyruvate decarboxylase (IPDC), an enzyme that catalyzes the conversion of indole-3-pyruvic acid to indole-3-acetaldehyde and is critical for indole-3-acetic acid (IAA; auxin) production. In this mutant, which is characterized by their null production of IAA, repression of primary root growth induced by the bacterium, as well as the formation of numerous lateral roots and long root hairs was not observed. Accordingly, the phenotype of plants inoculated with FAJ0009 was comparable to those cultivated on an axenic medium. The authors used transgenic plants expressing the auxin-inducible synthetic gene construct *DR5:GUS* to determine whether the interaction with *Azospirillum* could induce an auxin response. In fact, this was the case, as they were able to verify the induction of the auxin response in the root and foliage. This study demonstrated that auxin production was a major factor for *Azospirillum*-modulated root architecture configuration.

Recognizing the diversity of mechanisms involved in plant biostimulation by *Azospirillum*, it is expected that the degree of modification on developmental processes is associated with bacterial spread over the root, the secretion of phytohormones, or the emission of volatile compounds. Studies of the interaction between *A. brasilense* Sp-5 with *Arabidopsis* plants using the three different in vitro systems described above showed that reduced primary root growth, promotion of lateral root formation, and increased root hair growth are maximum upon bacterial root colonization (Figure 2). When the bacterium is inoculated at a certain distance from the root, the secretion of bioactive compounds (i.e., auxin) diffusing into the growth medium is sufficient to promote the growth of root hairs, while in the interaction using divided Petri plates that allow plant–bacteria communication solely through volatile compounds, the shortening of the taproot was not observed. In all three interaction systems, the bacterium stimulated plant biomass production.^20^

To determine whether bacterial colonization affects root growth by repressing cell division, elongation, or both, research has focused on the detailed analysis of meristems and cell elongation zones of the primary root.^39^ It showed that the induction of root hairs occurs due to the shortening of epidermal cells, while in the report by Méndez-Gómez et al.,^20^ both cell elongation and expression of the mitotic cyclin CYCB1 were reduced in relation to the time of bacterial inoculation, which also indicates an effect on mitosis. Cessation of primary root growth and cell elongation coincided with the activation of peroxidase and cell wall thickness, whereas peroxidase inhibition restored root growth upon *A. brasilense* inoculation,^39^ indicating that Reactive Oxygen Species (ROS) act as cellular messengers for auxin and elicitors mediating root growth adjustment to *Azospirillum.*

The successful recognition between roots and bacteria relies on diffusible molecules and cellular structural components. For bacteria harboring flagella, specific epitopes within flagellin act as elicitors for both plant defense and development.^40^^,^^41^ Direct contact of certain cellular components of *A. argentinense* with plant roots induces a growth response.^7^ For example, the *A. argentinense* REC3 flagellin can, on the one hand, protect strawberry plants against the fungus *Macrophomina phaseolina* by inducing biochemical, histological and molecular responses,^42^ and on the other hand, promote the development of wheat roots in plants growing in a rhizotron.^43^ Because the flagellin of the *Azospirillum* flagellum also acts as a microbe-associated molecular pattern (MAMP),^42^ it is possible that the *A. argentinense* Az39 flagellin is involved in signaling mechanisms that induce changes in root architecture, in addition to mechanisms mediated by phytohormones.

In a recent study, Mora et al.​​​​​​^21^ showed that for *Arabidopsis*, *A. argentinense* Az39 interaction, auxin is also an important factor for root architecture remodeling, but it is not unique since an auxin-deficient mutant caused by deletion of the gene encoding IPDC has a mild effect towards inhibiting root growth and promoting lateral root formation. Purified flagellin from Az39 strain had a dose-dependent effect on root hair development, which suggests that the overall root morphology and absorptive volume attributed to the direct physical contact between the root and bacterial cells could depend on flagellin as a signaling molecule (Figure 2), contributing to a more developed root system due to an increased root hair formation.

 Root exudates contain both nutritious and bioactive components that affect bacterial behavior. Nisha et al.^44^ found that cytidine, a pea seedling exudate and *Azospirillum brasilense* Sp7T increased both the number and length of pea roots and the bacterium could be found as part of the epiphytic root microbiome. These data are promising towards formulation of plant biostimulants based on plant exudates and *Azospirillum* spp.

### Nutrient acquisition

**Figure 3.**
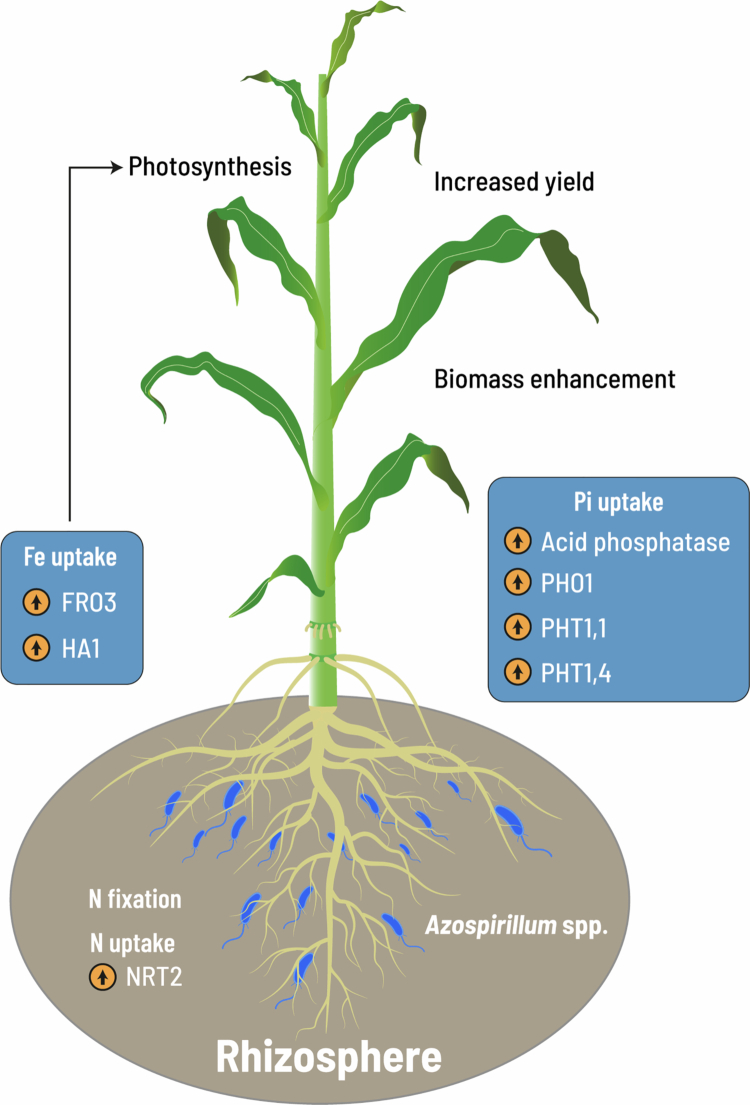
* Azospirillum* enhances plant biomass through an improved nutrition. Nitrogen (*N*) fixation is a prime mechanism attributed to the beneficial effects of the bacterium and is also related to the expression of the nitrate transporter NRT2. Several genes involved in phosphorus acquisition, including acid phosphatase enzyme, help to solubilize *P* from organic sources, whereas PHO1, PHT1,1, and PHT1,4 proteins help plants to acquire more available phosphate when inoculated with *Azospirillum*. In inoculated plants, the ferric chelate reductases FRO3 and HA1 ATPase improve iron nutrition and photosynthesis.

Plant nutrition in modern intensive agriculture is achieved by applying large amounts of chemical fertilizers, which are costly in terms of monetary investments and environmental impact. *Azospirillum* fixes nitrogen and thus, inoculated plants can access biologically available *N* forms but also acquire other essential nutrients by inducing root branching or, as demonstrated for phosphorus and iron, inducing molecularly their mechanisms for acquisition (Figure 3).

#### Nitrogen

Among all nutrients required by plants, nitrogen is the most demanded since it plays a primary role in achieving optimal growth and a higher yield in crops, horticultural species, and fruit trees. Nitrogen is the largest component of atmospheric air; for this reason, the potential of *Azospirillum* to convert elemental *N* into ammonia has been one of the most interesting and widely studied traits since its identification in 1925 by M.W. Beijerinck. This property was initially the major mechanism related to the plant growth-promoting capacity of *Azospirillum* and still shows promise to ameliorate *N* status in many plant families.^45–48^

Recent findings show that seed inoculation with *A. brasilense* Ab-V5 increased the growth of maize under *N* limitation through the modulation of biochemical and physiological processes related to better plant growth.^49^ Similarly, enhanced potato plant growth in response to *Azospirillum* strains characterized as nitrogen fixers correlated with higher nitrogen content in plants, an effect associated with nitrogen-fixing and improved nitrogen use efficiency, nitrogen uptake, and better nitrogen utilization.^50^ These effects are consistent with the modulation of transcriptional responses associated with the uptake, assimilation, and efficient use of nitrogen.^51^ Growth promotion by *A. brasilense* in *Arabidopsis* correlates with the expression of NRT2 genes, which encode membrane proteins for nitrate uptake and transport.^39^ The way in which the bacterium influences the uptake of fixed *N* and its distribution within the root and towards the foliage is under study.

#### Phosphorus

Phosphorus is required in high amounts to support plant growth and productivity owing to its important function as a structural component of macromolecules and the energetic reactions of cells. Most soils, particularly alkaline and acidic soils, suffer from ***P*** deficiency, which can be corrected by fertilizer application.^52^ Unlike the numerous reports on *Azospirillum* and nitrogen nutrition over the years, there are fewer reports on other mineral nutrients. However, a few recent reports indicate that *Azospirillum* spp. can influence phosphorus availability through the solubilization of insoluble phosphates and the efficiency of plants to take up this nutrient.^53^^,^^54^

Phosphate deprivation in *Arabidopsis* triggers the so-called low Pi rescue system, which consists of the formation of short roots with increased exploratory and absorptive potential, anthocyanin accumulation in leaves, and the induction of genes for organic acid and phosphatase biosynthesis and exudation.^55^ Interestingly, in the interaction of *Arabidopsis* plants with *A. brasilense* under low phosphate availability, the bacterium could alleviate stress symptoms such as growth inhibition and anthocyanin production, besides increasing the phosphate content. These responses were associated with a higher phosphorus solubilization capacity; upregulated expression of key genes in signaling for phosphorus uptake, such as *PHT1;1, PHT1;4, PHO1*, and *miR399*; and increased root hair density and length.^54^ Comparable results in *Arabidopsis* were obtained with the *A. brasilense* Sp7 strain, where the formation of branched root systems occurred regardless of the Pi dose applied, and also involved regulation of genes encoding components of the low-Pi rescue system, such as *PHT1;9, PHO1,* and *miR399.*^53^

#### Iron

Iron is an important nutrient with roles in respiration and photosynthesis and acts as a cofactor of many enzymes. Thus, leaf yellowing is a typical symptom of cereal plants and fruit trees growing on iron-deficient soils such as alkaline soils.^56^ The application of *A. brasilense* to cucumber plants increased Fe accumulation and alleviated Fe chlorosis symptoms through an increased Fe reduction and rhizosphere acidification. The bacterium induced genes encoding Fe-chelate reductase (*CsFRO*) and PM H^+^-ATPase (*CsHA1*) and enhanced Fe acquisition under both Fe-sufficient and Fe-limiting conditions.^57^^,^^58^ Scott et al.^59^ using ion chromatography with chemiluminescence detection, showed that *A. brasilense* HM053 increases yield by 30%–50% and four-fold Fe content of maize compared to non-inoculated plants.

### Hormonal regulation of plant growth

The production of phytohormones including auxins, cytokinins, gibberellins, and abscisic acid, plays an important role in plant growth promotion by *Azospirillum.*^60–63^ Whether these regulators are released to reinforce cross-kingdom plant-bacteria recognition or simply because they are integral components for the bacterial cell-to-cell communication remains to be clarified.

#### Auxin

Auxins are a group of molecules mainly represented by indole-3-acetic acid (IAA), which orchestrates plant growth and development.^64^ IAA production appears to be a desirable signature of plant growth-promoting rhizobacteria acting as a driver for lateral root formation, and certainly is a major factor by which *Azospirillum* increases plant biomass production.^60^^,^^62^^,^^65^^,^^66^

Auxin production by *A. brasilense* Sp245 led to reduced primary root growth and increased lateral root and root hair formation, which were not detected in plants inoculated with *A. brasilense* FAJ0009 mutant.^22^ Regulation of root architecture and plant growth promotion mediated by auxin was also reported in the *A. brasilense* Sp245–*Arabidopsis* interaction performed by Méndez-Gómez et al.^20^ ,using different interaction systems. More recently, a mild effect of an auxin-producing mutant Az39 *ipdC*- of *A. argentinense* on root system architecture unveiled strain-specific effects, which can be magnified by root perception of flagellin.^21^

#### Cytokinin

Along with auxins, cytokinins are molecules with a major role in regulating plant growth and development from seed germination to senescence through directly controlling cell cycle transitions.^67^^,^^68^ Since the discovery of cytokinin-like molecules produced by *Azospirillum*, these phytohormones have been suggested to mediate the plant responses induced by these bacteria.^69^ Consistently, *A. brasilense* RA-17, a zeatin-producing strain, but not *A. brasilense* RA-18, which does not produce zeatin, promoted the growth and yield of wheat and reinforced the antioxidant system.^70^ In addition, *Arabidopsis* biostimulation by *A. brasilense* Sp245 depends on the cytokinin signaling pathway since during the interaction, the expression of two molecular markers of the cytokinin response, *TCS:GFP* and *ARR5:GUS*, was induced, and knockout mutants of the genes encoding cytokinin receptors showed reduced effects of this bacterium on root growth.^20^

#### Abscisic acid

The production of abscisic acid by *A. brasilense* Sp. 245 occurs under standard growth conditions but is augmented upon NaCl supplementation. In *A. thaliana* seedlings, bacterial inoculation enhanced two-fold the ABA content, which accounts for the performance of inoculated plants under adverse environmental conditions such as salt or drought.^71^ In a recent study, Degon et al.​​​^72^ correlated the growth of rice plants and biomass production under high salt concentrations with the expression of genes modulated by ABA amongst other phytohormones, which may account for the reinforcement of the antioxidant system and ion and nutrient transport. These findings help to understand the contribution of *Azospirillum* to protect plants from abiotic stress through bacterial ABA production and inducing ABA signaling in plant hosts.

### Lipopolysaccharides

Lipopolysaccharides (LPS) are structural components of the outer membrane of bacteria that protect cells from environmental stressors, confer drug resistance, and contribute to pathogenesis and symbiosis.^73–76^ These macromolecules are regarded as microbe-associated molecular patterns (MAMPs), which are recognized by the plant innate immune system and induce plant defense via ROS and nitric oxide acting as second messengers.^77^

The application of LPS from *A. brasilense* Sp245 to wheat plants increased the growth and accelerated the life cycle of plants without significantly affecting grain yield.^73^ The effects of LPS were also determined in *Arabidopsis* plants both *in vitro* and in soil, and in each system, LPS increased growth and fruit yield, responses that were related to activation of TOR kinase signaling.^75^

Comparison of the bioactivity of LPS from *A. brasilense* with those from the plant pathogen *P. aeruginosa* PAO1 revealed a differential response in wheat seedlings, in which only the molecules of the beneficial bacteria increased total plant length and total fresh weight.^77^ LPS extracts from either bacterium increased peroxidase activity and hydrogen peroxide (H_2_O_2_) content in the root; however, the LPS from the pathogenic bacterium generated higher increments, indicating that it is the ROS balance, rather than their maximum accumulation that triggers root morphogenesis.

LPS from *A. brasilense* Sp-5 promoted callus formation in wheat and their mitotic potential and stimulated the regeneration of cultured tissues.^78^ When sprayed on plants grown in soil, these molecules increased the size of leaves, fruits, and seeds at the completion of the life cycle, which correlated with more branched and robust stems and roots, and induction of TOR signaling, a critical pathway orchestrating growth and metabolism.^79^

## Molecular plant responses to *Azospirillum*

### Genomic and proteomic signatures

Transcriptomic and proteomic analyses have greatly contributed to understanding the functions of genes and proteins associated with metabolism, signaling, and transport in the plant–*Azospirillum* interaction. Camilios-Nieto et al. (2014), using an RNA-seq transcriptional analysis of wheat roots inoculated with *A. brasilense*, found 776 genes differentially expressed. Fifty genes were related to the expression of microRNAs such as MIR444, which was upregulated and controls root growth and development, nitrate signaling, brassinosteroid biosynthesis, and resistance to pathogens.^80–82^ Other regulated genes encode disease resistance proteins contributing to the immune response, the SMALL AUXIN UP RNA (SAUR) genes that control growth and developmental processes in response to auxin, and 1-aminocyclopropane−1-carboxylic acid (ACC) oxidase that converts ACC to ethylene.^83–85^

Besides transporting water, nutrients, and energy molecules, the vascular system serves as a communication route that distributes hormones, peptides, proteins, and RNAs associated with environmental sensing from the root to the shoot and vice versa.^86^ The regulation of gene expression in plants by *Azospirillum* is not limited to root cells but also occurs in the shoot system. Tomato plants exhibited 243 genes upregulated and 184 downregulated in the foliage 35 d after root inoculation with *A. baldaniorum.*^87^ Most regulated genes were implicated in photosynthesis, water and nutrient transport, water stress, and ROS homeostasis. Camilios-Neto et al.^88^ reported that root cells colonized by *A. brasilense* FP2 had downregulated *ETTIN/ARF3*, an auxin-sensitive transcription factor that mediates gynoecium development. The expression of this regulator of reproductive maturation in the root suggests that ETTIN/ARF3 could be long-distance mobilized within the plant.

 Adaptive molecular responses to *Azospirillum* are dynamic and change according to several factors, including the interaction time.^89^ During the first 24 h of interaction, plants exhibit changes in the expression of genes involved in defense and abiotic stress responses. Afterward, in an intermediate phase, between 2 and 8 d post-inoculation, many of these genes were downregulated. However, in late periods of interaction, bacterial sensing still occurs, and genes involved in defense and growth are upregulated.^89^

Rice plants inoculated with *A. brasilense* Sp7 exhibited 1,622 regulated genes (490 upregulated and 1,132 downregulated) on the first interaction day and 1,995 genes (619 upregulated and 1,376 downregulated) after 14 d of interaction. However, 300 genes were regulated in both periods.^90^ Gene ontology analysis showed that the majority of regulated genes on the first day were related to metabolism, response to stimuli, response to stress, and catalytic activity. The most enriched category by day 14 was cellular components. These data show a clear difference in the plant gene expression during the early stages of interaction, when a recognition process is initiated, versus the late stages, when the bacteria spread, create a biofilm and establish themselves within the root. Besides, *A. brasilense* Sp7 led to differential host protein accumulation depending on the inoculation time in two different plant species. The first sampling period was where the greatest changes in protein abundance occurred, mainly in those associated with metabolism and redox homeostasis.^91^

The genetic diversity and plasticity of plants allow each species to interact and respond to microorganisms in a specific way due to their particularities in phenotypic traits and molecular mechanisms.^92^^,^^93^ For example, tomato and maize plants inoculated with *Azospirillum brasilense* Sp7 showed differences in protein expression. Disease resistance proteins were accumulated in tomato plants but not in maize, and an opposite trend occurred with the pathogenic cell lysis proteins.^91^

Different proteomic profiles were exhibited by crop varieties and not only among distinct species in response to *Azospirillum* interaction. Drogue et al.^94^ showed unique transcriptomic fingerprints in two rice varieties (Cigalon and Nipponbare) after inoculation with *A. lipoferum* 4B or *A. lipoferum* B510. The authors observed altered expression of 1,243 and 2,141 genes in Cigalon and Nipponbare varieties, respectively, by strain 4B. On the other hand, strain B510 modified the expression of 3,865 genes in Cigalon and 2539 in Nipponbare. Most of the regulated genes were related to primary metabolism, transport, transcriptional regulation, and protein fate. The authors further showed that the regulation of plant-specific genes is also influenced by the specific strain of *Azospirillum* applied.

### Target of rapamycin signaling

Plants, being sessile organisms, have developed fine mechanisms that allow them to integrate various environmental signals to respond and adapt to their environment, either by modifying their metabolism, growth, development, and/or defense.^92^^,^^95^ The main enzymes that carry out the transduction of environmental stimuli are protein kinases, a family of phosphotransferases that can transfer phosphate groups to a wide variety of downstream molecular targets such as transcription factors.^96^

The TORC1 complex, composed of the Ser/Thr TOR kinase and the subunits LST8 and Raptor, acts as a sensor of nutrients, energy molecules, hormones, and external signals that impact gene expression, and protein synthesis and activity.^97^ A target of the TORC1 complex is S6 kinase (S6K), which promotes protein translation by phosphorylation of S6 (RPS6) protein.^98^ The E2Fa/E2Fb transcription factors are also activated by TOR to induce root growth and leaf formation.^99^ Besides, TOR regulates cell division in the root meristem and in leaves and plant responses to abiotic stress, involving auxin sensing, and inhibiting abscisic acid receptors, respectively.^97^^,^^99^

Several traits from *Azospirillum* may contribute to TOR signaling, root bacterization for instance enhanced expression of TOR kinase in shoot and root meristems and promoted phosphorylation of its downstream target the p70 ribosomal S6 kinase (S6K) that modulates protein synthesis and ribosomal biogenesis.^79^ The TOR inhibitor AZD-5 inhibited plant growth and cell division in root meristems and in lateral root primordia, interfering with the phytostimulation by *A. brasilense*, and consistently, the *A. brasilense* mutant FAJ009, impaired in auxin production, was unable to elicit TOR signaling to the level observed for the wild-type strain, showing the importance of this phytohormone to stimulate TOR signaling. The above information suggests that by means of producing auxin and/or its direct root colonization and detection of LPS by still unknown receptors in plant cells, *Azospirillum* recognition induces TOR signaling within plant tissues.

## Concluding remarks

The global need to increase food production makes necessary the incorporation of effective management strategies, such as the use of beneficial bacteria that can contribute to saving inputs, money, and reducing environmental pollution, and at the same time increases crop production over planted extensions. In this sense, the multiple properties of *Azospirillum* offer alternatives to reduce the application of fertilizers, strengthen the immune system of plants and support the photosynthetic and reproductive organs through improved rooting. The aspects that should advance *Azospirillum* knowledge for the coming years are: (1) To explore the variability of phytostimulation mechanisms in bacteria of different origins since environmental factors adjust the adaptive properties of organisms and their interactions with other biological groups. (2) To analyze the contribution of quorum-sensing systems, particularly those involving *N*-acyl-*L*-homoserine lactones and cyclodipeptides in bacteria-plant recognition and plant growth stimulation. (3) To test bacterial consortia, including *Azospirillum*, in combination with other probiotic isolates in model plants and crops. (4) Identify and overexpress select genes in the bacterium to increase their phytostimulant properties, and (5) advance in the formulation of broad-spectrum biotechnological products for application in the field. The years to come will show the promise of rhizosphere engineering beyond *Azospirillum* and the potential of the microbiomes as a plant breeding strategy.
